# A prehabilitation-enhanced nomogram for predicting early pulmonary recovery failure after lung tumor surgery: development and multicenter validation

**DOI:** 10.3389/fmed.2026.1842606

**Published:** 2026-07-13

**Authors:** Sanhua Lian, Xihua Lian, Zhixing Zhu, Chunping Shi, Fengyu Chen

**Affiliations:** 1 Department of Cardiothoracic Surgery, Joint Logistics Support Force No. 910 Hospital, Quanzhou, China; 2 Department of Ultrasound Medicine, Second Clinical Medical School of Fujian Medical University, Quanzhou, China; 3 Department of Sleep Medicine, Second Clinical Medical School of Fujian Medical University, Quanzhou, China

**Keywords:** lung tumor surgery, nomogram, postoperative pulmonary complications, prehabilitation, risk prediction

## Abstract

**Objectives:**

To develop and validate a practical prehabilitation-enhanced nomogram for predicting early postoperative pulmonary recovery failure within 7 days after lung tumor surgery.

**Methods:**

In this multicenter retrospective study, patients were enrolled from the first center as the development cohort and the second center as the external test cohort. Routine perioperative variables and quantified 14-day prehabilitation process indicators were collected. A routine-variable model and a prehabilitation-enhanced model were compared under the same framework. Independent predictors were identified by multivariable logistic regression and integrated into a nomogram. Model performance was evaluated in terms of discrimination, calibration, and clinical utility.

**Results:**

Adding prehabilitation indicators improved discrimination in the training, internal validation, and external test cohorts, with the area under the curve increasing from 0.946 to 0.972, from 0.825 to 0.863, and from 0.861 to 0.883, respectively. The final nomogram included five predictors: intraoperative blood loss, DLCO% predicted, preoperative resting SpO_2_, incentive spirometry target-achieved days (0–14), and breathing training target-achieved days (0–14). The nomogram showed good discrimination with AUCs of 0.921 (95% CI 0.891–0.952), 0.885 (95% CI 0.826–0.943), and 0.845 (95% CI 0.778–0.912) in the training, internal validation, and external test cohorts, respectively, and outperformed any single predictor. Calibration was acceptable across cohorts, with Brier scores of 0.097, 0.122, and 0.143, and decision curve analysis supported clinical usefulness across relevant threshold probabilities.

**Conclusion:**

A prehabilitation-enhanced nomogram enables individualized early risk stratification for early postoperative pulmonary recovery failure within 7 days and may support targeted prevention and nursing resource allocation.

## 1 Introduction

Lung tumor resection remains a cornerstone of curative treatment, but early postoperative pulmonary complications and delayed pulmonary recovery are still common and clinically costly, leading to prolonged hospitalization, higher resource use, and increased downstream morbidity (1, 2). Recent evidence suggests that postoperative pulmonary complications after lung cancer surgery occur at a non-negligible rate and are strongly influenced by baseline pulmonary reserve, chronic obstructive pulmonary disease (COPD), smoking history, and perioperative factors, yet many of these risks evolve dynamically during the perioperative window and may not be fully captured by static preoperative assessments alone (3).

In parallel, prehabilitation, particularly programs combining exercise training, respiratory training, and behavior support, has gained momentum as a potentially modifiable pathway to improve postoperative outcomes (4). Systematic reviews and meta-analyses indicate that preoperative exercise and respiratory training can reduce overall postoperative complications in patients undergoing lung resection, although intervention designs and adherence vary substantially across studies (5). More recent studies have extended prehabilitation to multimodal, structured pathways and to virtual formats to improve accessibility and continuity, supporting feasibility and potential outcome benefits in real-world thoracic surgery settings (6–8).

Meanwhile, risk prediction for post-operative pulmonary complications has grown rapidly, but most models are retrospective, often lack robust external validation, and rarely incorporate modifiable perioperative process indicators. Most tools target in-hospital or 30-day complications and longer-term respiratory outcomes after resection (9), whereas models focused on very early pulmonary recovery impairment within the first postoperative week remain relatively scarce despite its relevance to bedside nursing decisions. A recent systematic review identified numerous prediction models but highlighted persistent limitations in transportability and validation that constrain adoption (10, 11). Notably, models that integrate conventional clinical and perioperative variables with prehabilitation process or adherence metrics are still uncommon, although preoperative “what the patient actually does” may add incremental value beyond baseline physiology alone (5, 6).

Therefore, this study aimed to develop and validate a prehabilitation-enhanced nomogram for predicting early postoperative pulmonary recovery failure within 7 days (EPRF-7d) after lung tumor surgery. This tool is intended to support early risk stratification and guide targeted preventive nursing and intensified prehabilitation/rehabilitation for patients at high risk, thereby helping to reduce progression to severe pulmonary complications.

## 2 Methods

### 2.1 Study design and participants

This was a multicenter prediction-model study designed to develop and validate a prehabilitation-enhanced nomogram for estimating the risk of early postoperative pulmonary recovery failure within 7 days after lung tumor resection. The development cohort (for model development and internal validation) was derived from inpatients who underwent lung tumor resection in the Department of Thoracic Surgery, from the first center while an independent cohort from the Department of Thoracic Surgery, The from the second center served as the external validation cohort. Patients operated between January 2020 and December 2025 were eligible. The study was conducted in accordance with the Declaration of Helsinki and was approved by the Ethics Committee (2026–30 and 2026–60). All data were de-identified prior to analysis.

Eligible patients met the following criteria: (1) age 18 years or older; (2) underwent elective lung tumor surgery with pulmonary parenchymal resection at a participating center, including wedge resection, segmentectomy, or lobectomy; (3) had a traceable 14-day preoperative prehabilitation window with available documentation to calculate prehabilitation process indicators; (4) had sufficient perioperative records to reliably adjudicate the primary endpoint (EPRF-7d); (5) had core clinical variables available for modeling, including baseline characteristics, pulmonary function results, surgical approach and procedure, and intraoperative records. Patients were excluded if they (1) did not undergo lung tumor resection; (2) underwent emergency or unplanned surgery that precluded assessment of the 14-day prehabilitation window; (3) underwent major combined thoracic surgery (chest wall/diaphragm resection or reconstruction, major airway reconstruction, or esophageal surgery); (4) had an outcome that could not be reliably adjudicated from traceable records; (5) had missingness in key predictors that precluded analysis; (6) had major data integrity issues. All participants provided written informed consent.

### 2.2 Definition and quantification of prehabilitation process indicators

The prehabilitation window was uniformly defined as the 14 days before surgery. Seven quantified, traceable prehabilitation process indicators were extracted using standardized definitions:

(1) Psychological support sessions (times): number of documented psychological support and counseling encounters delivered by specialized nurses or the nursing team during the 14-day window.
(2) Peer meeting participation (times): number of documented peer-support and peer-education sessions attended.
(3) Family training completion (Yes/No): completion status of required family training content, documented by training records/signatures.
(4) Incentive spirometry target-achieved days (0–14): number of days within the 14-day window meeting a predefined daily target standard based on the unified Standard Operating Procedure (SOP) (Supplementary Materials 1).
(5) Breathing training target-achieved days (0–14): number of days meeting daily breathing training targets based on the unified SOP (Supplementary Materials 2).
(6) Mean daily steps (steps/day): average daily steps over the 14-day window, derived from device export using a harmonized calculation approach (total steps/recorded days).
(7) Relaxation training completed days (0–14): number of days completing standardized relaxation training based on the unified SOP (Supplementary Materials 3).

To enhance comparability across centers, the data source and the “target achieved/completion” criteria were standardized and implemented consistently.

### 2.3 Outcome definition (EPRF-7d)

The primary endpoint, early postoperative pulmonary recovery failure within 7 days (EPRF-7d), was defined as a composite outcome. An event was recorded (coded as 1; otherwise 0) if any of the following occurred within 7 postoperative days:

(1) Escalation of respiratory support: defined as escalation beyond routine postoperative care documented by medical orders, including initiation of high-flow nasal cannula, non-invasive ventilation, re-intubation, or invasive mechanical ventilation, and/or escalation of oxygen delivery mode due to hypoxemia or increased work of breathing.
(2) Failure of secretion clearance: defined as the need for therapeutic bronchoscopy for mucus plugging and retained secretions associated with clinical deterioration such as worsening oxygenation and documented in procedure notes and medical records.
(3) Failure of lung re-expansion: defined as atelectasis or incomplete lung re-expansion confirmed on chest radiography or CT that required additional clinical intervention beyond routine postoperative care, such as therapeutic bronchoscopy, escalation of ventilatory support, application of positive end-expiratory pressure, continuous positive airway pressure, or other documented lung re-expansion measures.
(4) Pulmonary infection: defined as clinically diagnosed postoperative pneumonia within 7 days supported by compatible imaging findings plus clinical and/or laboratory evidence and requiring targeted antimicrobial therapy.

Outcome adjudication was based on traceable evidence from medical charts, orders, bronchoscopy records, imaging reports, respiratory support documentation, and medication records, following a prespecified adjudication SOP across centers.

### 2.4 Candidate predictors

Candidate predictors were prespecified and grouped into four modules: (1) preoperative baseline characteristics and comorbidities, including age, gender, height, weight, body mass index, smoking history, primary lung diagnosis, comorbid COPD, and history of prior thoracic surgery; (2) preoperative physiological and functional status and pulmonary function, including preoperative resting peripheral oxygen saturation (SpO_2_), preoperative respiratory rate, preoperative cough effectiveness score, St George's Respiratory Questionnaire total score (SGRQ), Hospital Anxiety and Depression Scale total score (HADS), 6-min walk distance (6MWD), forced expiratory volume in 1 second percent predicted (FEV1% predicted), and diffusing capacity of the lung for carbon monoxide percent predicted (DLCO% predicted); (3) perioperative surgical factors, including surgical approach, surgical procedure type, operation duration, intraoperative blood loss, and intraoperative transfusion; and (4) quantified prehabilitation process indicators recorded during the 14-day preoperative window, including psychological support sessions, peer-support meetings, family or caregiver training completion, days meeting prespecified targets for incentive spirometry and breathing training, and other prespecified prehabilitation adherence measures captured in the study protocol. Continuous predictors were kept on their original continuous scales whenever feasible. All data were extracted from electronic medical records, anesthesia and operative notes, laboratory and imaging systems, bronchoscopy and procedure records, and nursing and prehabilitation documentation at both centers.

### 2.5 Cohort splitting and modeling strategy

Within the development cohort, patients were randomly split into a training cohort and an internal validation cohort at a 7:3 ratio using outcome-stratified sampling to preserve the event proportion of EPRF-7d. Two prediction models were developed under an identical modeling framework. The base model included routine clinical and perioperative predictors and excluded prehabilitation indicators. The prehabilitation-enhanced model incorporated the seven quantified prehabilitation process indicators in addition to the base predictors. The incremental value of adding prehabilitation indicators was evaluated by comparing model discrimination, calibration, and clinical utility. After confirming improved performance with the prehabilitation-enhanced model, it was finalized as the primary model, internally validated, and then applied to the external test cohort from the Second Affiliated Hospital of Fujian Medical University using fixed coefficients for independent validation.

### 2.6 Statistical analysis

All analyses were performed in R (R Foundation for Statistical Computing, Vienna, Austria), with data preprocessing in Microsoft Excel. Continuous variables were assessed for normality (Shapiro–Wilk and histograms) and variance homogeneity (Levene's test). Normally distributed data were presented as mean ± SD and compared with the independent-samples t test; non-normal data as median (IQR) and compared with the Mann–Whitney *U*-test. Categorical variables were presented as n (%) and compared using the chi-square or Fisher's exact test, as appropriate.

Candidate predictors were prespecified based on clinical relevance and prior literature. In the training cohort, univariable logistic regression was used to screen associations with EPRF-7d, followed by multivariable logistic regression to identify independent predictors, reported as odds ratios (95% CIs). Multicollinearity was assessed using variance inflation factor (VIF) and tolerance (VIF > 5 or tolerance <0.2). Independent predictors were used to construct a nomogram.

Discrimination was evaluated by ROC curves and AUC (95% CI) in the training, internal validation, and external test cohorts; AUCs were compared using the DeLong test. The optimal cutoff was determined by the Youden index, and performance was summarized. Calibration was assessed with calibration plots, Brier score, and the Hosmer–Lemeshow test. Internal validation used 1,000-bootstrap resampling to obtain optimism-corrected estimates. Clinical utility was evaluated using decision curve analysis and clinical impact curves. Two-sided *P* < 0.05 was considered statistically significant.

## 3 Results

### 3.1 Patient inclusion of the study

In the development cohort, 638 patients were screened and 113 were excluded, including age younger than 18 years (*n* = 22), non–lung tumor surgery (*n* = 39), major concurrent thoracic combined procedures (*n* = 14), unassessable 14-day preoperative prehabilitation window (*n* = 19), and missing key clinical variables (*n* = 19). Overall, 525 patients met the eligibility criteria, and 13 withdrew for personal reasons, leaving 512 patients for analysis. These patients were randomly split into the training cohort (*n* = 359) and internal validation cohort (*n* = 153) using a 7:3 ratio.

**Figure 1:**
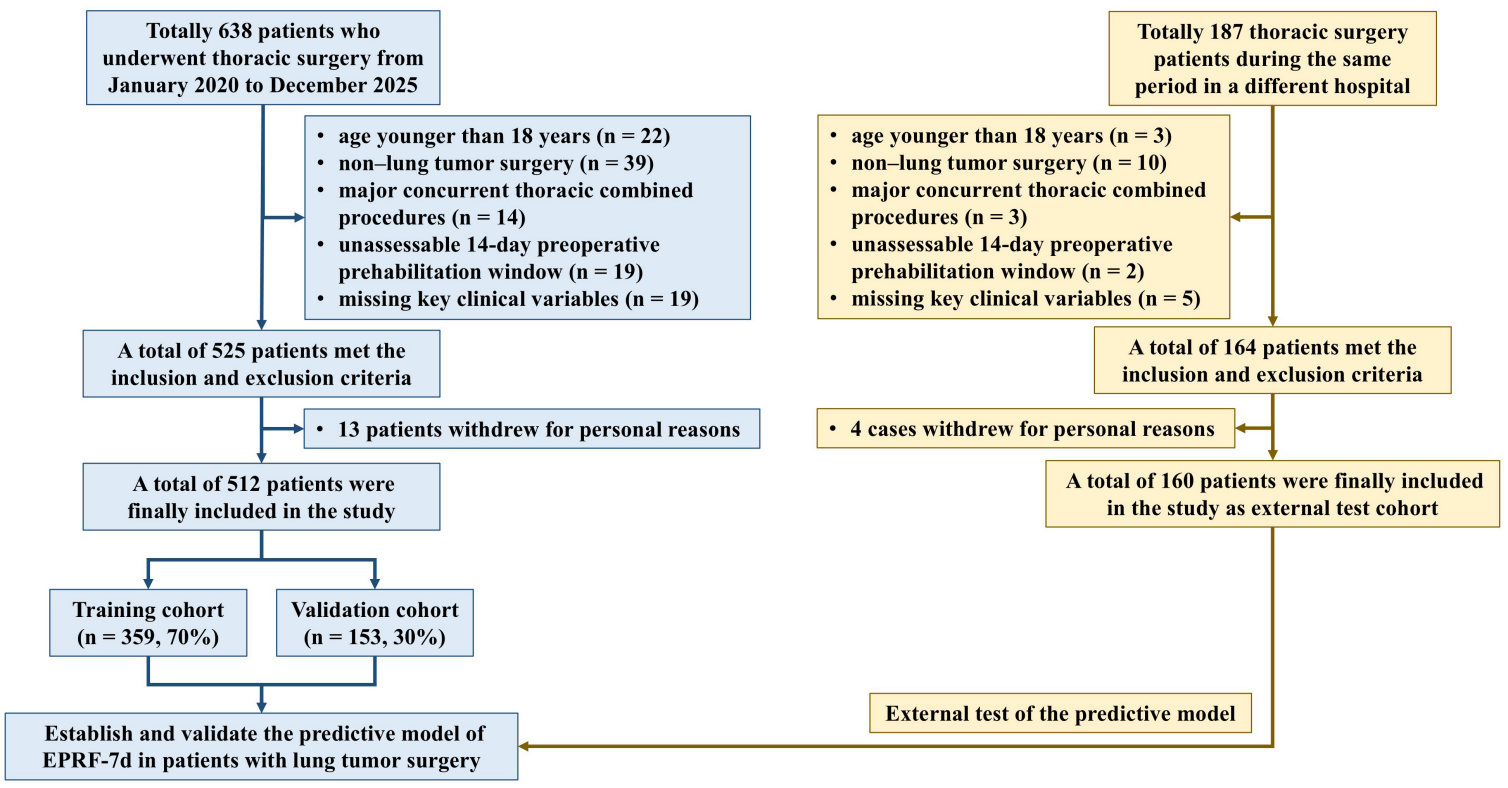
Flow diagram of patient inclusion. EPRF-7d: early postoperative pulmonary recovery failure within 7 days.

At the second center, 187 patients were screened and 23 were excluded, including age younger than 18 years (*n* = 3), non–lung tumor surgery (*n* = 10), major concurrent thoracic combined procedures (*n* = 3), unassessable 14-day preoperative prehabilitation window (*n* = 2), and missing key clinical variables (*n* = 5). In total, 164 eligible patients were enrolled; 4 withdrew for personal reasons, resulting in 160 patients in the external test cohort. The patient selection process is presented in Figure 1.

### 3.2 Baseline comparability of the training and validation cohorts

Baseline characteristics of the training and internal validation cohorts were summarized in Table 1. Overall, these two cohorts were well balanced with respect to demographics, pulmonary function measures, preoperative physiological and functional status, perioperative surgical variables, and preoperative 14-day prehabilitation process and adherence indicators, with no statistically significant between-group differences observed across the assessed variables (all *P* > 0.05). These findings suggest that the random split did not introduce meaningful baseline imbalance and provided a robust basis for subsequent model development and internal validation.

**Table 1:** Baseline characteristics of the training and validation cohorts.

Characteristic	Training (*n* = 359)	Validation (*n* = 153)	*P* value
EPRF-7d, *n* (%)			
No	244 (68.0%)	102 (66.7%)	0.774
Yes	115 (32.0%)	51 (33.3%)	
Age, years	51.259 ± 19.307	53.804 ± 18.816	0.166
**Gender**, ***n*** **(%)**			
Female	189 (52.6%)	74 (48.4%)	0.375
Male	170 (47.4%)	79 (51.6%)	
BMI, kg/m^2^	23.903 ± 4.765	24.287 ± 4.901	0.414
Smoking, *n* (%)			0.945
No	210 (58.5%)	89 (58.2%)	
Yes	149 (41.5%)	64 (41.8%)	
**Primary diagnosis**, ***n*** **(%)**			0.840
Adenocarcinoma	171 (47.6%)	75 (49.0%)	
Benign or non-tumor	149 (41.5%)	64 (41.8%)	
Squamous cell carcinoma	39 (10.9%)	14 (9.2%)	
**COPD**, ***n*** **(%)**			0.945
No	210 (58.5%)	90 (58.8%)	
Yes	149 (41.5%)	63 (41.2%)	
FEV1%pred	84.733 ± 15.009	84.719 ± 14.064	0.992
DLCO%pred	89.273 ± 14.453	89.052 ± 14.311	0.874
**Prior thoracic surgery**, ***n*** **(%)**			0.939
No	182 (50.7%)	77 (50.3%)	
Yes	177 (49.3%)	76 (49.7%)	
SpO_2_, %	96.412 ± 3.393	96.046 ± 3.644	0.289
Respiratory rate, breaths/min	17.019 ± 2.398	17.131 ± 2.489	0.640
**Cough effectiveness score (0–3)**, ***n*** **(%)**			0.659
0	101 (28.1%)	36 (23.5%)	
1	97 (27.0%)	40 (26.1%)	
2	75 (20.9%)	35 (22.9%)	
3	86 (24.0%)	42 (27.5%)	
SGRQ, total score	9.435 ± 9.331	8.843 ± 9.396	0.514
HADS, total score	9.153 ± 3.988	8.915 ± 3.704	0.516
6MWD, m	499.159 ± 28.202	497.516 ± 29.847	0.563
**Surgical approach**, ***n*** **(%)**			
RATS	99 (27.6%)	32 (20.9%)	0.236
Thoracotomy	70 (19.5%)	29 (19.0%)	
VATS	190 (52.9%)	92 (60.1%)	
**Surgical procedure**, ***n*** **(%)**			
Lobectomy	136 (37.9%)	55 (35.9%)	0.467
Segmentectomy	118 (32.9%)	45 (29.4%)	
Wedge resection	105 (29.2%)	53 (34.6%)	
Operation time, min	139.443 ± 40.515	134.699 ± 41.849	0.237
Blood loss, mL	185.992 ± 142.357	173.431 ± 126.579	0.323
Drainage 24 h, mL	295.485 ± 115.162	282.353 ± 106.704	0.214
Prehab incentive spirometry days (0–14)	6.783 ± 4.295	6.869 ± 4.234	0.833
Prehab breathing training days (0–14)	7.028 ± 4.200	7.405 ± 3.845	0.324
Prehab psychological support sessions, *n*	2.908 ± 1.946	2.765 ± 1.989	0.453
**Prehab peer meeting, times [*****n*** **(%)]**			0.957
0	122 (34.0%)	50 (32.7%)	
1	107 (29.8%)	46 (30.1%)	
2	130 (36.2%)	57 (37.3%)	
**Prehab Family training**, ***n*** **(%)**			0.499
No	176 (49.0%)	80 (52.3%)	
Yes	183 (51.0%)	73 (47.7%)	
Prehab steps, steps/day	3,820.173 ± 1,953.571	3,599.588 ± 1,886.510	0.232
Prehab relaxation training days (0–14)	6.292 ± 3.778	6.706 ± 4.083	0.285

Values are presented as mean ± SD for continuous variables and n (%) for categorical variables. BMI, body mass index; COPD, chronic obstructive pulmonary disease; DLCO, diffusing capacity of the lung for carbon monoxide; EPRF-7d, early postoperative pulmonary recovery failure within 7 days; FEV1, forced expiratory volume in 1 s; HADS, Hospital Anxiety and Depression Scale; Prehab, prehabilitation; RATS, robot-assisted thoracic surgery; SGRQ, St George's Respiratory Questionnaire; SpO_2_, peripheral oxygen saturation; 6MWD, 6-min walk distance; VATS, video-assisted thoracoscopic surgery.

### 3.3 Incremental value of incorporating prehabilitation indicators

**Figure 2:**
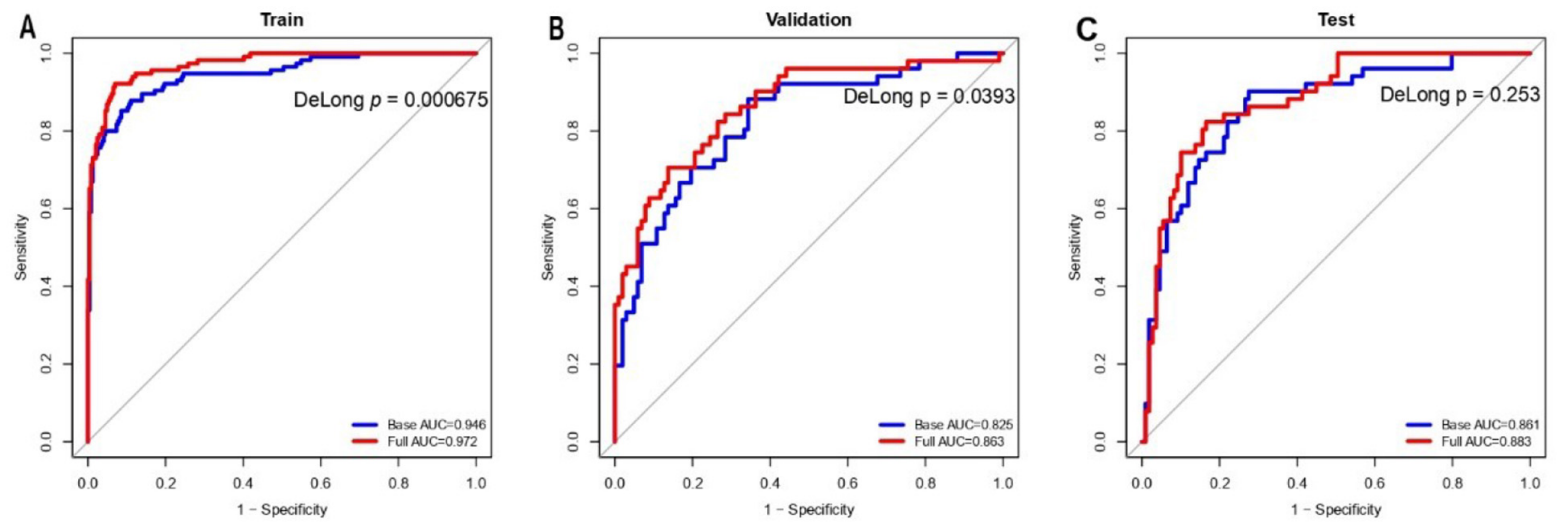
Receiver operating characteristic (ROC) curves comparing the Base model and Full model across cohorts. ROC curves for discriminating postoperative early pulmonary recovery failure were plotted for the Base model (without prehabilitation variables) and the Full model (with preoperative 14-day prehabilitation process/compliance variables). **(A)** Training cohort; **(B)** validation cohort; **(C)** cohort. The AUCs for Base vs. Full were 0.946 vs. 0.972 in training (*P* < 0.001), 0.825 vs. 0.863 in internal validation (*P* < 0.05), and 0.861 vs. 0.883 in external validation (*P* = 0.253).

Using the same modeling framework, we developed a base model that excluded preoperative prehabilitation indicators and a full model that additionally incorporated quantified prehabilitation process and completion measures over the 14-day preoperative window. Discrimination of the two models was compared across the training cohort, internal validation cohort, and external test cohort. As shown in Table 2 and Figure 2, the full model consistently yielded higher area under the curve (AUC) values than the base model in all three cohorts. Specifically, the AUC increased from 0.946 to 0.972 in the training cohort, with a statistically significant difference by the DeLong test (*P* < 0.001), and from 0.825 to 0.863 in the internal validation cohort (*P* < 0.05). In the external test cohort, the area under the curve increased from 0.861 to 0.883 (*P* = 0.253), supporting the incremental predictive value of prehabilitation process information.

**Table 2:** Discriminative performance of the Base and Full models across the training, internal validation, and external test cohorts.

Cohort	AUC-Base (95% CI)	AUC-Full (95% CI)	*P* value
Train (359)	0.946 [0.920, 0.972]	0.972 [0.957, 0.987]	<0.001
Validation (153)	0.825 [0.755, 0.896]	0.863 [0.799, 0.926]	0.0393
Test (160)	0.861 [0.799, 0.924]	0.883 [0.829, 0.937]	0.253

AUC, area under the curve; CI, confidence interval.

On the basis of this incremental benefit, subsequent analyses focused on the predictors retained in the full model to construct an interpretable nomogram intended for bedside use, enabling individualized risk estimation and risk stratification for early postoperative pulmonary recovery failure.

### 3.4 Univariable logistic regression analysis in the training cohort

In the training cohort (*n* = 359), EPRF-7d was used as the dependent variable, and each candidate predictor was examined using univariable logistic regression to screen potential risk factors. Indicators reflecting pulmonary reserve and oxygenation status were significantly associated with EPRF-7d. Higher DLCO% predicted was associated with a lower risk, and higher preoperative resting SpO_2_ also showed a protective association. From a perioperative perspective, greater intraoperative blood loss was associated with an increased risk of EPRF-7d. In addition, comorbid COPD was associated with a higher risk of EPRF-7d. In addition, a greater number of peer-support meetings, more days meeting pre-specified targets for incentive spirometry, more days meeting pre-specified targets for breathing training, and higher mean daily steps were each associated with a lower risk of EPRF-7d (Table 3).

**Table 3:** Univariate and multivariable logistic regression analysis for EPRF-7d in the training cohort.

Predictor	Univariate analysis		Multivariable analysis		VIF	Tolerance
	OR (95% CI)	*P* value	OR (95% CI)	*P* value		
Age, years	0.994 (0.983–1.006)	0.349				
Gender (male vs. female)	0.795 (0.509–1.242)	0.313				
Smoking (yes vs. no)	1.170 (0.750–1.824)	0.489				
BMI, kg/m^2^	1.002 (0.956–1.050)	0.933				
FEV1%pred	1.005 (0.990–1.021)	0.480				
DLCO%pred	0.918 (0.898–0.939)	<0.001	0.924 (0.895–0.952)	<0.001	1.073	0.932
SpO_2_, %	0.748 (0.694–0.807)	<0.001	0.755 (0.660–0.854)	<0.001	1.208	0.828
RR, breaths/min	0.991 (0.903–1.087)	0.841				
Cough score (0–3)	1.085 (0.893–1.320)	0.411				
SGRQ total score	1.010 (0.986–1.034)	0.416				
HADS total score	0.978 (0.924–1.034)	0.433				
6MWD, m	1.008 (1.000–1.016)	0.055				
Primary diagnosis	1.150 (0.714–1.851)	0.565				
COPD (yes vs. no)	5.137 (3.182–8.292)	<0.001				
Prior thoracic surgery (yes vs. no)	1.184 (0.760–1.846)	0.455				
Surgical approach	1.617 (0.817–3.201)	0.168				
Surgical procedure	0.387 (0.224–0.669)	<0.001				
Operation time, min	0.997 (0.992–1.003)	0.350				
Blood loss, mL	1.007 (1.006–1.009)	<0.001	1.009 (1.007–1.013)	<0.001	1.181	0.847
Drainage, mL	1.000 (0.998–1.002)	0.725				
Prehab psychological support, *n*	1.036 (0.925–1.161)	0.539				
Prehab peer meeting, *n*	0.711 (0.543–0.930)	0.013				
Prehab incentive spirometry days (0–14)	0.864 (0.817–0.915)	<0.001	0.803 (0.724–0.882)	<0.001	1.103	0.907
Prehab breathing training days (0–14)	0.863 (0.814–0.914)	<0.001	0.827 (0.752–0.902)	<0.001	1.068	0.936
Prehab steps, per 1,000 steps/day	0.839 (0.744–0.946)	0.004				
Prehab relaxation days (0–14)	0.965 (0.909–1.024)	0.235				
Prehab family training (yes vs. no)	1.189 (0.763–1.854)	0.445				

ORs for continuous variables are reported per 1-unit increase (as defined by the recorded unit). Variables labeled as “coded” were analyzed using the original dataset coding and should be interpreted with caution. BMI, body mass index; CI, confidence interval; COPD, chronic obstructive pulmonary disease; DLCO%pred, diffusing capacity of the lung for carbon monoxide (% predicted); EPRF-7d, early postoperative pulmonary recovery failure within 7 days; FEV1%pred, forced expiratory volume in 1 s (% predicted); HADS, Hospital Anxiety and Depression Scale; OR, odds ratio; Prehab, prehabilitation; RR, respiratory rate; SGRQ, St George's Respiratory Questionnaire; SpO_2_, peripheral oxygen saturation; 6MWD, 6-min walk distance; VIF: variance inflation factor.

### 3.5 Multivariable logistic regression analysis in the training cohort

In the multivariable logistic regression model fitted in the training cohort, measures of pulmonary reserve and oxygenation, perioperative surgical burden, and several preoperative prehabilitation adherence indicators remained independently associated with EPRF-7d, as summarized in Table 3 (all *P* < 0.05). Overall, higher diffusing capacity of DLCO% predicted and higher preoperative resting SpO_2_ were associated with a lower risk of EPRF-7d. More complete execution of prehabilitation activities, including incentive spirometry training, breathing training, and walking activity, also showed independent protective associations, whereas greater intraoperative blood loss was independently associated with increased risk (Table 3).

### 3.6 Multicollinearity assessment

Multicollinearity diagnostics for variables retained in the multivariable model indicated no evidence of problematic collinearity. The adjusted generalized variance inflation factor values were close to 1 (1.068–1.208) and the tolerance values remained high (0.828–0.936). With all variance inflation factors remaining well below 5, there was no evidence of meaningful multicollinearity, suggesting that the predictors were not overly correlated and that the model coefficients were estimated reliably (Table 3).

### 3.7 Establishment of nomogram model and predictive formula

**Figure 3:**
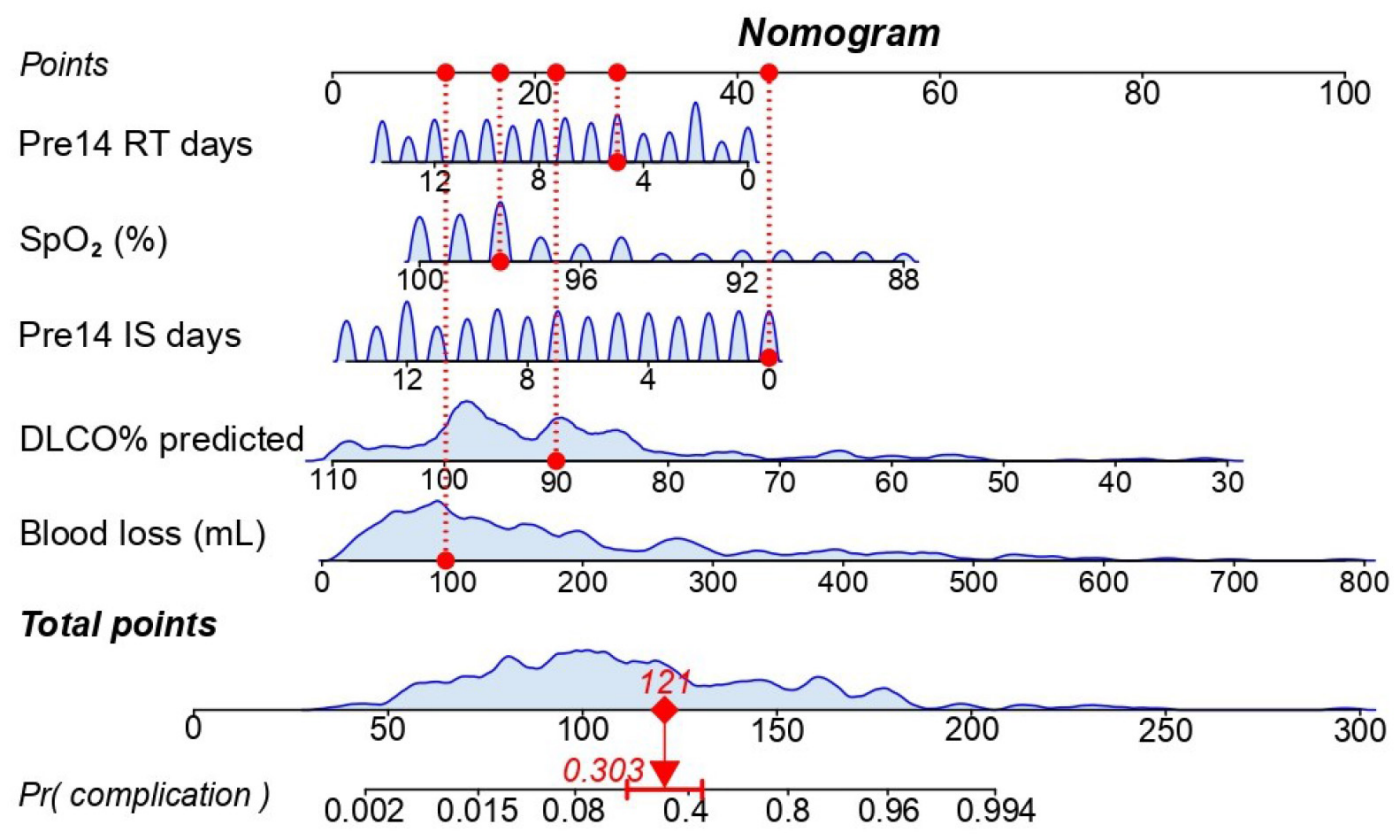
Nomogram for predicting early postoperative pulmonary recovery failure within 7 days (EPRF-7d). The nomogram integrates five independent predictors identified by multivariable logistic regression, including intraoperative blood loss (mL), diffusing capacity of the lung for carbon monoxide percent predicted (DLCO% predicted), preoperative resting peripheral oxygen saturation (SpO_2_, %), incentive spirometry target-achieved days during the 14-day preoperative window (Pre14 IS days), and breathing training target-achieved days during the 14-day preoperative window (Pre14 RT days). For each predictor, the patient's value is located on its corresponding axis and projected to the “Points” scale to obtain a score; the scores are summed to yield “Total points”, which is then mapped to the predicted probability of EPRF-7d.

A nomogram model was developed based on the independent predictors identified in the final multivariable logistic regression analysis. The model incorporated blood loss (mL), DLCO% predicted, preoperative SpO_2_ (%), prehabilitation incentive spirometry days (0–14), and prehabilitation breathing training days (0–14). Each predictor was assigned a point value proportional to its regression coefficient, and the sum of points corresponded to the predicted probability of EPRF-7d. The graphical representation of the nomogram is shown in Figure 3, illustrating the relative contribution of each predictor to overall risk estimation. All predictors were entered on their original scales (blood loss per 1 mL; DLCO%pred and SpO_2_ per 1%; prehabilitation variables per 1 day).

The final logistic regression formula for predicting EPRF-7d was:

Logit (P_EPRF − 7d_) = 33.458583 + 0.008997 × blood loss – 0.077227 × DLCO%pred – 0.208359 × prehab incentive spirometry days – 0.278522 × SpO_2_ – 0.180284 × prehab breathing training days

The predicted probability was then calculated as:

P (EPRF-7d) = 1 / [1 + exp(–logit (P_EPRF − 7d_))]

In this model, higher blood loss was associated with an increased risk of EPRF-7d, whereas higher DLCO% predicted, higher preoperative SpO_2_, and greater adherence to prehabilitation incentive spirometry and breathing training (more days achieving targets during the preoperative 14-day period) were protective.

Worked example. For the case in the training dataset (blood loss = 95 mL; DLCO%pred = 90%; preoperative SpO_2_ = 98%; prehabilitation incentive spirometry days = 0; prehabilitation breathing training days = 5), the calculated logit (P_EPRF − 7d_) was −0.834, yielding a predicted probability of *P* (EPRF-7d) ≈ 0.303 (Figure 3).

### 3.8 Evaluation of model performance

#### 3.8.1 Discrimination and diagnostic accuracy

**Figure 4:**
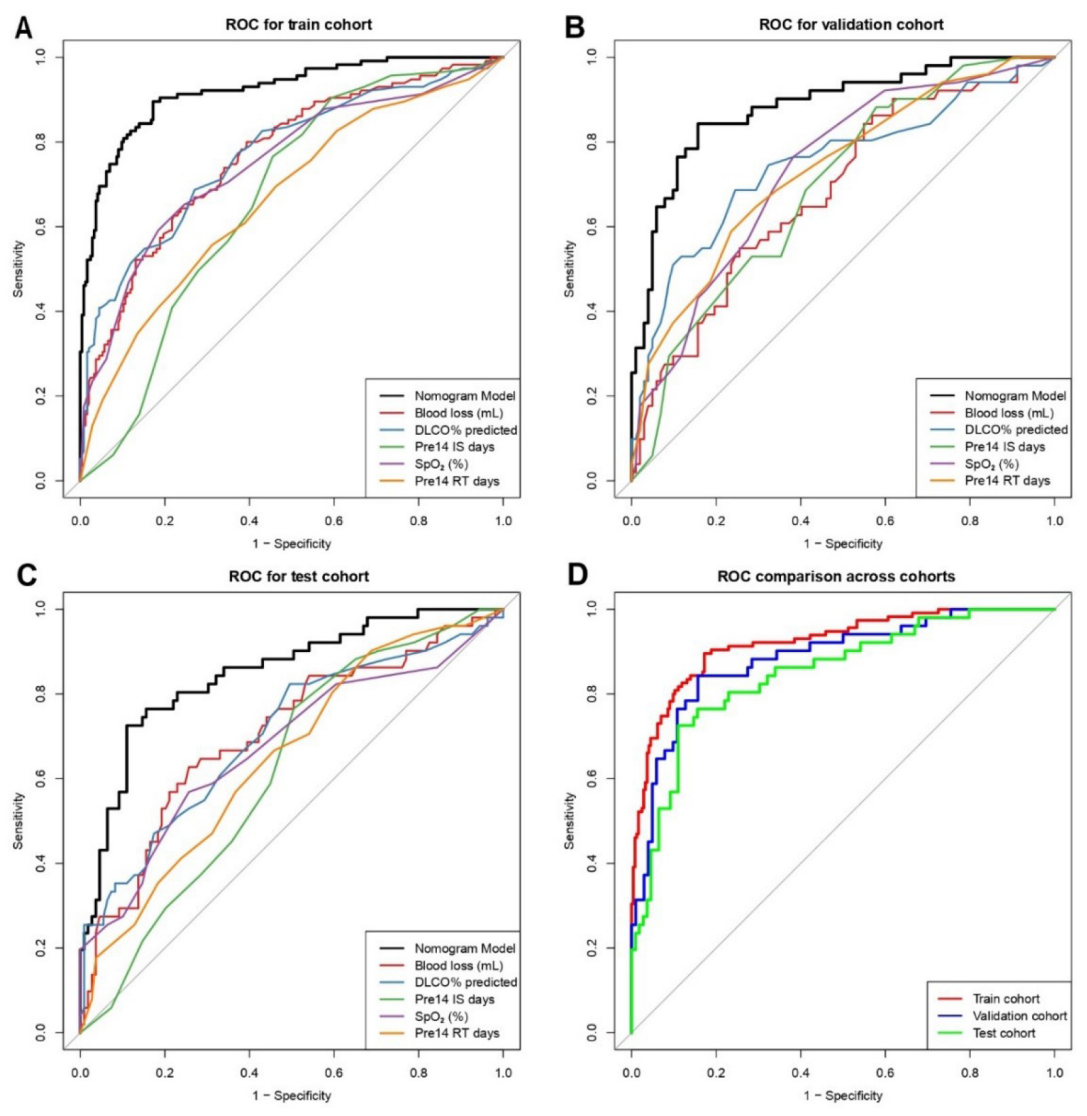
ROC curves of the nomogram model and individual predictors in the training, validation, and test cohorts. Receiver operating characteristic curve analysis was performed to evaluate the discriminative performance of the nomogram model and its independent predictors, including intraoperative blood loss (mL), diffusing capacity of the lung for carbon monoxide percent predicted (DLCO% predicted), preoperative resting peripheral oxygen saturation (SpO_2_, %), incentive spirometry target-achieved days during the 14-day preoperative window (Pre14 IS days), and breathing training target-achieved days during the 14-day preoperative window (Pre14 RT days) in the training cohort **(A)**, internal validation cohort **(B)**, and external test cohort **(C)**. Panel **(D)** compares the nomogram model across cohorts. The diagonal line indicates no discrimination with an area under the curve of 0.5. AUC, area under the curve; ROC, receiver operating characteristic.

EPRF-7d was treated as a binary outcome (1 = events and 0 = non-events). The final nomogram model was evaluated using ROC analysis in the training, internal validation, and external test cohorts (Table 4; Figure 4), yielding AUCs of 0.921 (95% CI 0.891–0.952), 0.885 (95% CI 0.826–0.943), and 0.845 (95% CI 0.778–0.912), respectively, indicating good discrimination across datasets. DeLong comparisons further showed the nomogram achieved significantly higher AUCs than any single predictor, supporting the added value of integrating perioperative factors with prehabilitation process indicators (all *P* < 0.01; Figure 4). AUCs were not significantly different between cohorts by DeLong comparisons, suggesting stable performance across cohorts.

**Table 4:** Diagnostic performance of the nomogram model for predicting EPRF-7d across datasets.

Cohort	Train (359)	Validation (153)	Test (160)
AUC (95% CI)	0.921 (0.891–0.952)	0.885 (0.826–0.943)	0.845 (0.778–0.912)
Cut-off	0.245	0.391	0.383
Youden index	0.724	0.686	0.613
Sensitivity	0.896	0.804	0.72
Specificity	0.828	0.882	0.893
PPV	0.71	0.774	0.766
NPV	0.944	0.9	0.868
PLR	5.203	6.833	6.742
NLR	0.126	0.222	0.313

Cut-offs were selected within each dataset by maximizing the Youden index; performance metrics are reported at the selected cut-off. AUC, area under the receiver operating characteristic curve; CI, confidence interval; EPRF-7d, early postoperative pulmonary recovery failure within 7 days; NLR, negative likelihood ratio; NPV, negative predictive value; PLR, positive likelihood ratio; PPV, positive predictive value.

Using the maximum Youden index to determine the optimal cutoff, the Youden index, sensitivity, and specificity were 0.724, 0.896, and 0.828 in the training cohort, 0.686, 0.804, and 0.882 in the internal validation cohort, and 0.613, 0.720, and 0.893 in the external test cohort, respectively (Table 4). Together, these findings indicate that the nomogram provides robust overall diagnostic performance and practical usability across cohorts.

#### 3.8.2 Calibration

Bootstrap internal validation (1,000 resamples) in the training cohort showed stable, optimism-corrected calibration (slope 0.948; intercept −0.035), and the corrected calibration curve closely tracked the ideal line (Supplementary Figure S1), indicating good agreement between predicted and observed risks.

**Figure 5:**
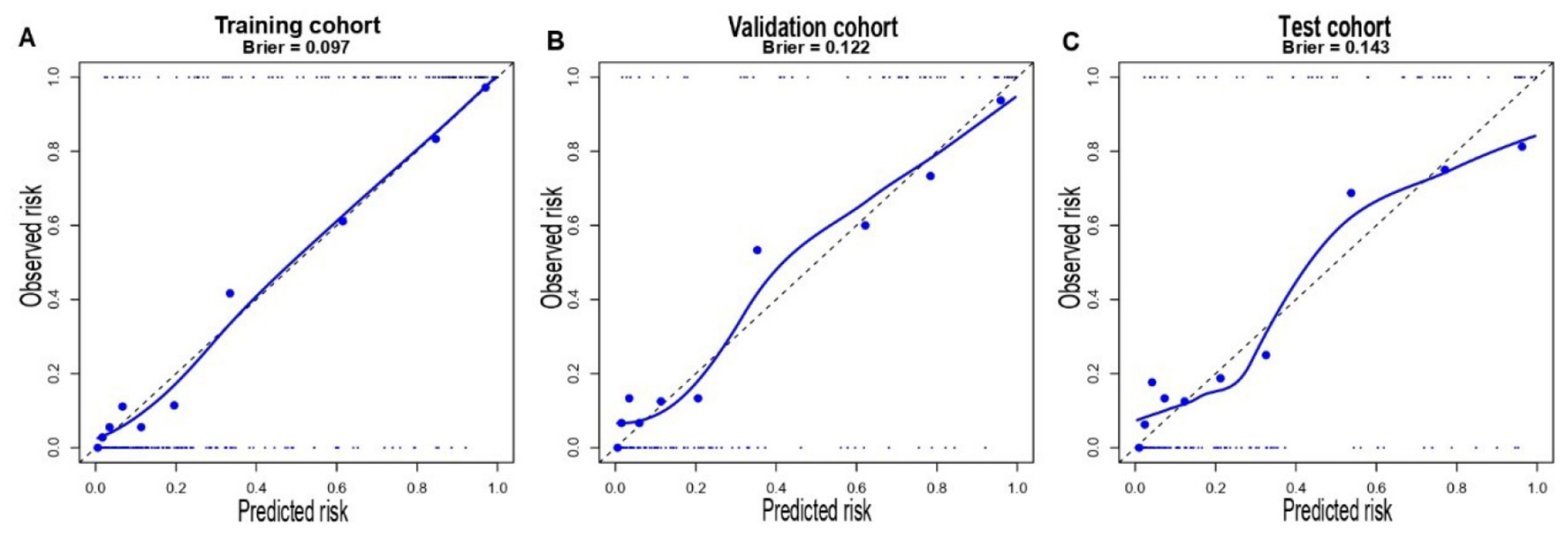
Calibration curves of the nomogram model across the training, validation, and test cohorts. Calibration plots are shown for the training **(A)**, internal validation **(B)**, and external test cohorts **(C)**, respectively. The x-axis denotes the predicted probability of early postoperative pulmonary recovery failure within 7 days, and the y-axis denotes the observed event rate. The dashed 45° line represents perfect calibration. Blue points indicate the observed event rates within grouped risk strata, and the blue smooth curve depicts the overall calibration trend.

Calibration was also consistent across cohorts, with Brier scores of 0.097 (training), 0.122 (internal validation), and 0.143 (external test) (Figure 5). Using a prespecified Hosmer–Lemeshow *P* > 0.05 as no evidence of poor fit, the model showed no significant miscalibration in the training cohort (χ^2^ = 5.862, *P* = 0.663) or internal validation cohort (χ^2^ = 10.255, *P* = 0.248), suggesting satisfactory fit without obvious overfitting or underfitting.

### 3.9 Clinical utility

#### 3.9.1 Decision curve analysis

**Figure 6:**
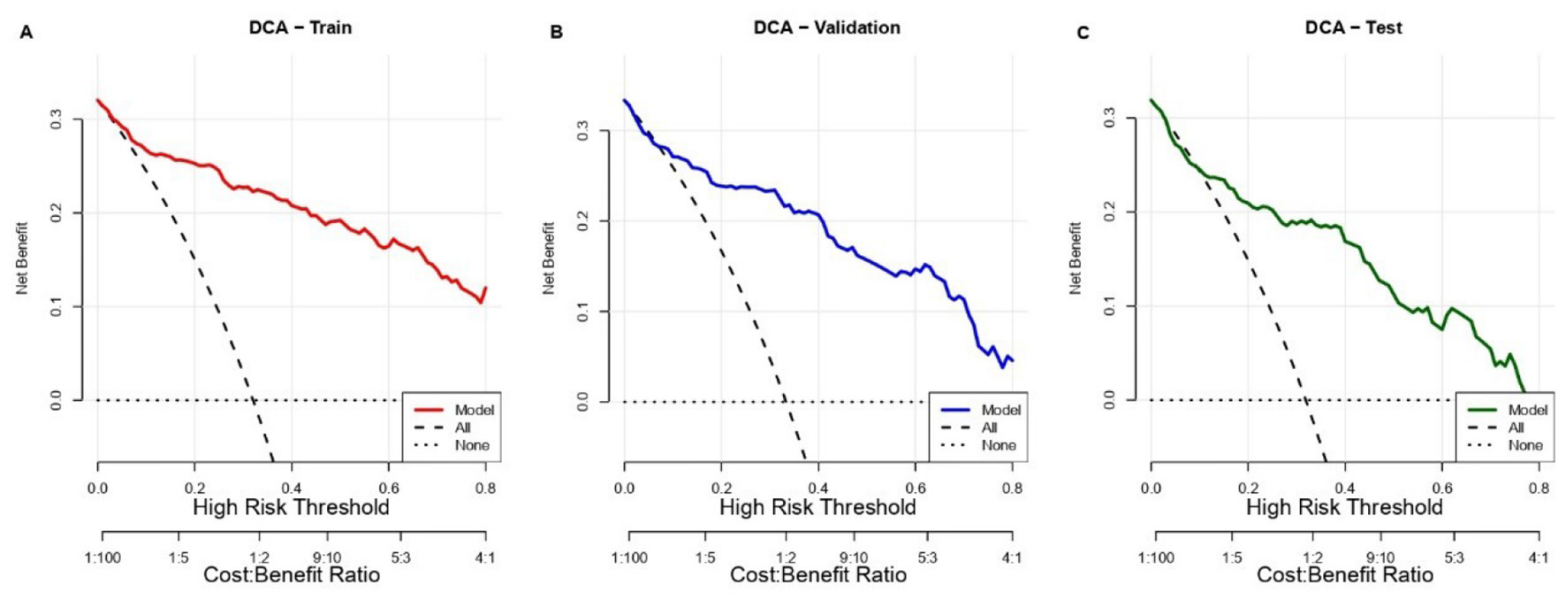
Decision curve analysis evaluating the clinical net benefit of the nomogram across cohorts. Decision curve analysis of the nomogram model for predicting EPRF-7d across the training **(A)**, internal validation **(B)**, and external test **(C)** cohorts. The nomogram provided a higher net benefit than the “treat-all” or “treat-none” strategies across a wide range of threshold probabilities in all three cohorts, indicating good clinical applicability and decision-making value of the model.

Decision curve analysis showed that the nomogram provided higher net benefit than treat-all and treat-none strategies across clinically relevant threshold probabilities in all cohorts (Figure 6). The net benefit across a wide range of threshold probabilities of approximately 0.03% to 80.0% in the training cohort (Figure 6A), about 6.2% to 80.0% in the internal validation cohort (Figure 6B), and approximately 9.3% to 80.0% in the external test cohort (Figure 6C), supporting its transportability and potential applicability in independent clinical settings.

#### 3.9.2 Clinical impact curve analysis

**Figure 7:**
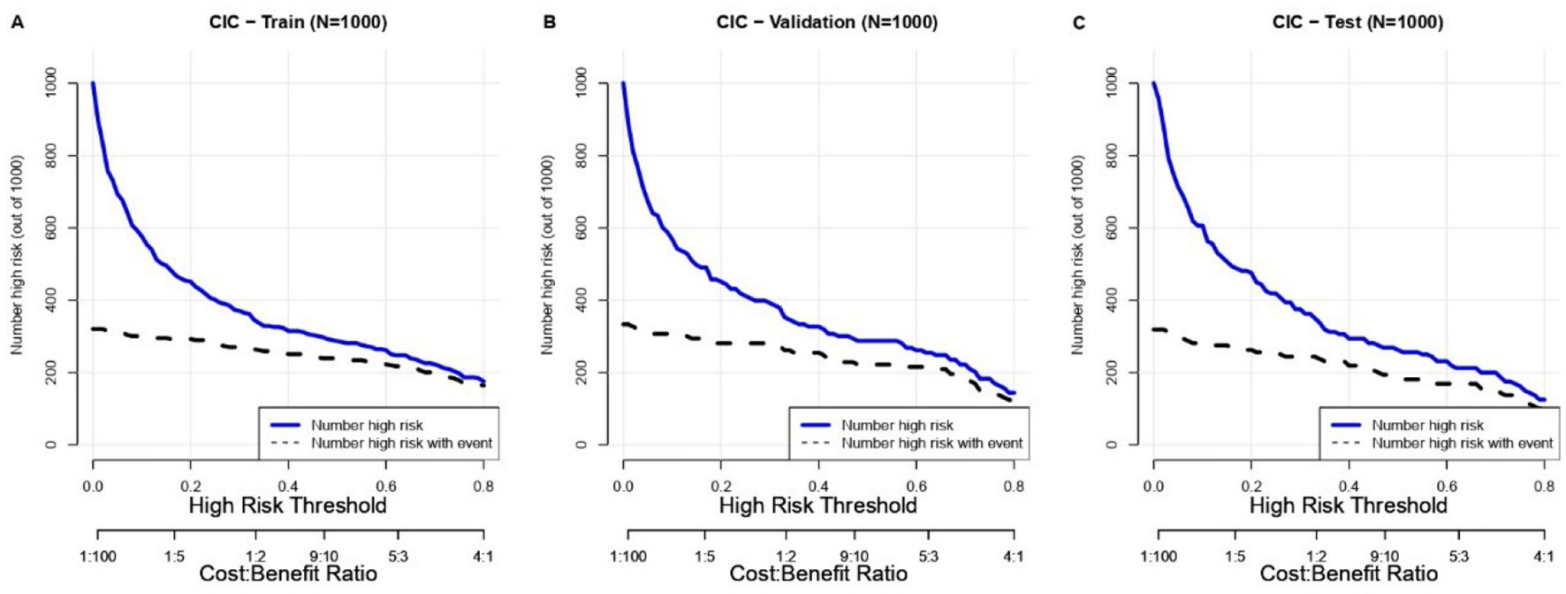
Clinical impact curves of the nomogram model in the training, internal validation, and external test cohorts. The clinical impact curve illustrates the expected number of patients classified as high risk (per 1,000 patients) across a range of threshold probabilities. The solid curve represents the number of high-risk patients identified by the model, and the dashed curve represents the number of high-risk patients who truly experienced EPRF-7d. The x-axis shows the threshold probability (with the corresponding cost: benefit ratio), and the y-axis shows the number of patients (out of 1,000). The separation between the two curves reflects the potential extent of unnecessary interventions, whereas closer alignment indicates higher event enrichment among those classified as high risk.

Clinical impact curve analysis was used to further illustrate the practical implications of applying the nomogram to identify patients at high risk of EPRF-7d across a range of threshold probabilities (Figure 7). Increasing the threshold probability led to a progressive decrease in the number of patients classified as high risk in all cohorts. In parallel, the number of patients classified as high risk who actually experienced EPRF-7d also decreased. Notably, at higher threshold probabilities of approximately 60% or above, the curve representing the predicted high-risk population approached the curve representing observed events among those deemed high risk, suggesting greater event enrichment and fewer false-positive classifications with potentially unnecessary interventions. Overall, the nomogram appears useful for high-risk screening and can support targeted intensification of prehabilitation nursing and perioperative resource allocation.

## 4 Discussion

This study developed and validated a prehabilitation-enhanced nomogram to predict early postoperative pulmonary recovery failure within 7 days (EPRF-7d) after lung tumor resection. Compared with a base model without prehabilitation indicators, the full model incorporating quantified 14-day prehabilitation process measures showed consistently better discrimination across the training, internal validation, and external test cohorts, supporting the added value of prehabilitation information. The final nomogram included five independent predictors, including blood loss, DLCO% predicted, preoperative resting SpO_2_, incentive spirometry target-achieved days, and breathing training target-achieved days, and demonstrated overall good discrimination, calibration, and clinical utility. These results suggest the nomogram may enable individualized risk stratification and guide targeted prehabilitation and perioperative resource allocation.

In this study, intraoperative blood loss emerged as an independent risk factor for EPRF-7d, likely reflecting greater surgical complexity and tissue trauma with heightened perioperative inflammatory stress and physiological instability, which are associated with postoperative pulmonary complications after lung resection (12, 13). Moreover, greater blood loss often prompts more intensive fluid resuscitation and, in some cases, transfusion; in lung resection patients, higher perioperative fluid administration has been linked to more pulmonary complications, plausibly via interstitial edema and impaired oxygenation that promote atelectasis and escalation of respiratory support (14). Consistently, even small-volume perioperative red blood cell transfusion has been associated with worse postoperative outcomes, supporting an integrated blood loss–resuscitation/transfusion–pulmonary vulnerability pathway to early recovery failure (15).

Higher DLCO% predicted was independently associated with a lower risk of EPRF-7d. DLCO% predicted reflects alveolar–capillary gas-transfer capacity and pulmonary vascular bed reserve and is a cornerstone of preoperative functional risk assessment in thoracic surgery (16). A reduced DLCO indicates impaired diffusion and/or loss of effective exchange surface, limiting tolerance to anesthesia, one-lung ventilation, and the postoperative reduction in gas-exchange area after lung resection (17), thereby predisposing patients to early oxygenation deterioration, secretion retention, and incomplete lung re-expansion (18). Accordingly, major guidelines recommend incorporating DLCO into perioperative risk stratification for lung resection candidates (17, 19, 20). Moreover, cohort data show that predicted postoperative DLCO strongly predicts pulmonary morbidity and operative mortality after lung resection (11, 21).

We found that higher preoperative resting SpO_2_ also showed a protective association with EPRF-7d, which is similar with previous study (22, 23). Preoperative resting SpO_2_ is a readily available, low-cost bedside indicator of oxygenation that can help capture limited baseline oxygen reserve and occult abnormalities in ventilation, diffusion, or ventilation–perfusion matching. Lower SpO_2_ may therefore signal reduced physiological tolerance to anesthesia, one-lung ventilation, and postoperative reductions in effective gas-exchange area, predisposing patients to early oxygen therapy escalation, atelectasis, and pulmonary infection even when conventional lung function values appear near operability thresholds (23). The clinical relevance of this marker is supported by the ARISCAT risk index, in which preoperative oxygen saturation is a core predictor for postoperative pulmonary complications, and by subsequent prospective external validation demonstrating the score's transportability across diverse surgical settings (24).

More incentive spirometry target-achieved days during the 14-day preoperative window was independently associated with a lower risk of EPRF-7d (25). Incentive spirometry promotes repeated sustained deep inspirations, which may improve alveolar recruitment and support secretion clearance, thereby reducing early events such as atelectasis and escalation of respiratory support (26). Importantly, using “target-achieved days” captures the *dose* of execution and adherence, a key determinant of clinical effectiveness; consistent with this, a thoracic-surgery study reported that preoperative incentive spirometry education improved postoperative compliance and was associated with fewer postoperative pulmonary complications (27).

In our study, more breathing training target-achieved days during the 14-day preoperative window was independently associated with a lower risk of EPRF-7d. Breathing training, often including inspiratory muscle training, breathing control, and effective coughing, may improve respiratory muscle endurance, ventilatory efficiency, and airway clearance, thereby reducing early events such as secretion retention and atelectasis (28, 29). This is consistent with evidence that structured, multimodal preoperative exercise and respiratory prehabilitation in lung cancer surgery is associated with fewer postoperative pulmonary complications and improved recovery (30).

This study focuses on early postoperative pulmonary recovery failure within 7 days, a clinically actionable window that aligns closely with perioperative nursing decision-making, and provides a bedside tool for individualized risk stratification. By moving beyond a simple prehabilitation delivered classification and quantifying prehabilitation as traceable process indicators incorporated into the prediction model, our approach enhances both the interpretability and the actionability of prehabilitation in routine care. Supported by multicenter development and validation together with discrimination, calibration, and clinical utility evaluations, the model offers a quantitative basis for perioperative resource allocation, risk-adapted follow-up, and targeted intensification of prehabilitation strategies.

Several limitations should be acknowledged. Although multicenter validation was performed, differences in patient case-mix and institutional nursing pathways may still limit generalizability. Prehabilitation process indicators rely on documentation and adherence assessment, which may introduce measurement error and between-center variability. In addition, EPRF-7d is a composite endpoint, and its components may not share identical mechanisms. Future work should include larger, prospective external validations, model updating with early postoperative dynamic measures, and risk-stratified interventional studies to determine whether model-guided intensification of prehabilitation and nursing care can improve clinical outcomes.

## 5 Conclusion

In conclusion, we developed and externally validated a practical prehabilitation-enhanced nomogram for predicting EPRF-7d after lung tumor surgery. By integrating routine perioperative factors with quantified prehabilitation process indicators, the model demonstrated good performance and potential clinical utility. This tool may facilitate early risk stratification and support targeted intensification of prehabilitation and perioperative nursing management to prevent early respiratory deterioration.

## Data Availability

The original contributions presented in the study are included in the article/Supplementary material, further inquiries can be directed to the corresponding author.

## References

[1] Baar W Semmelmann A Knoerlein J Weber F Heinrich S Loop T . Risk factors for postoperative pulmonary complications leading to increased in-hospital mortality in patients undergoing thoracotomy for primary lung cancer resection: a multicentre retrospective cohort study of the German thorax registry. J Clin Med. (2022) 11:5774. doi: 10.3390/jcm11195774 36233649 PMC9572507

[2] Im Y Park HY Shin S Shin SH Lee H Ahn JH . Prevalence of and risk factors for pulmonary complications after curative resection in otherwise healthy elderly patients with early stage lung cancer. Respir Res. (2019) 20:136. doi: 10.1186/s12931-019-1087-x 31272446 PMC6610954

[3] Deng T Song J Tuo J Wang Y Li J Ping Suen LK . Incidence and risk factors of pulmonary complications after lung cancer surgery: a systematic review and meta-analysis. Heliyon. (2024) 10:e32821. doi: 10.1016/j.heliyon.2024.e32821 38975138 PMC11226845

[4] Levett DZH Grocott MPW. Prehabilitation: impact on postoperative outcomes. Int Anesthesiol Clin. (2025) 63:68–76. doi: 10.1097/AIA.0000000000000481 40323728 PMC12144543

[5] Gravier FE Smondack P Prieur G Medrinal C Combret Y Muir JF . Effects of exercise training in people with non-small cell lung cancer before lung resection: a systematic review and meta-analysis. Thorax. (2022) 77:486–96. doi: 10.1136/thoraxjnl-2021-217242 34429375

[6] Mania K Pieczynska A Hojan K. Comprehensive multimodal prehabilitation for lung cancer: a systematic review of randomized controlled trials. Ther Clin Risk Manag. (2025) 21:1735–45. doi: 10.2147/TCRM.S563218 41426234 PMC12717860

[7] Mao JJ Molena D Desai K Baser RE Seluzicki C Rocco G . Participation in virtual prehabilitation and outcomes following thoracic cancer surgery. JAMA Netw Open. (2024) 7:e244084. doi: 10.1001/jamanetworkopen.2024.4084 38546649 PMC10979307

[8] Brat K Sova M Homolka P Plutinsky M Genzor S Pokorna A . Multimodal prehabilitation before lung resection surgery: a multicentre randomised controlled trial. Br J Anaesth. (2025) 135:188–96. doi: 10.1016/j.bja.2025.03.018 40374400 PMC12597589

[9] Verkoulen K Franssen A Vissers YLJ Hulsewe KWE Degens J Brecheisen R . The role of body composition and pulmonary function in predicting long-term survival after lung cancer surgery. Clin Nutr ESPEN. (2025) 68:140–7. doi: 10.1016/j.clnesp.2025.04.009 40349844

[10] Huang Z Han Y Zhuang H Jiang J Zhou C Yu H. Prediction models for postoperative pulmonary complications: a systematic review and meta-analysis. Br J Anaesth. (2025) 135:1415–27. doi: 10.1016/j.bja.2025.04.025 40473567 PMC12597335

[11] Chen S Deng T Yang Q Li J Shen J Luo X . Development and validation of an explainable machine learning model for predicting postoperative pulmonary complications after lung cancer surgery: a machine learning study. EClinicalMedicine. (2025) 86:103386. doi: 10.1016/j.eclinm.2025.103386 40791887 PMC12337024

[12] Yao L Wang W. Effect of intraoperative blood loss on postoperative pulmonary complications in patients undergoing video-assisted thoracoscopic surgery. Turk Gogus Kalp Damar Cerrahisi Derg. (2021) 29:347–53. doi: 10.5606/tgkdc.dergisi.2021.20657 34589253 PMC8462118

[13] Gigliotti S Guerriero G Mazza G Garofalo E Pavia G Amaddeo A . Perioperative blood biomarkers of infectious and non-infectious postoperative pulmonary complications: a narrative review. J Clin Med. (2026) 15:699. doi: 10.3390/jcm15020699 41598637 PMC12841655

[14] Arslantas MK Kara HV Tuncer BB Yildizeli B Yuksel M Bostanci K . Effect of the amount of intraoperative fluid administration on postoperative pulmonary complications following anatomic lung resections. J Thorac Cardiovasc Surg. (2015) 149:314–20, 21 e1. doi: 10.1016/j.jtcvs.2014.08.071 25304302

[15] Ferraris VA Davenport DL Saha SP Bernard A Austin PC Zwischenberger JB. Intraoperative transfusion of small amounts of blood heralds worse postoperative outcome in patients having noncardiac thoracic operations. Ann Thorac Surg. (2011) 91:1674–80. doi: 10.1016/j.athoracsur.2011.01.025 21514923

[16] Johnson DC. Interpretation of diffusing capacity. Chest. (2021) 159:2513–4. doi: 10.1016/j.chest.2020.12.054 34099143

[17] Donington J Ferguson M Mazzone P Handy J. Jr. , Schuchert M, Fernando H, et al. American college of chest physicians and society of thoracic surgeons consensus statement for evaluation and management for high-risk patients with stage I non-small cell lung cancer. Chest. (2012) 142:1620–35. doi: 10.1378/chest.12-0790 23208335

[18] Brunelli A Kim AW Berger KI Addrizzo-Harris DJ. Physiologic evaluation of the patient with lung cancer being considered for resectional surgery: diagnosis and management of lung cancer, 3rd ed: American college of chest physicians evidence-based clinical practice guidelines. Chest. (2013) 143:e166S–e90S. doi: 10.1378/chest.12-2395 23649437

[19] Spyratos D Zarogoulidis P Porpodis K Angelis N Papaiwannou A Kioumis I . Preoperative evaluation for lung cancer resection. J Thorac Dis. (2014) 6:S162–6. doi: 10.3978/j.issn.2072-1439.2014.03.06 24672690 PMC3966163

[20] Ohde Y Ueda K Okami J Saito H Sato T Yatsuyanagi E . Guidelines for preoperative pulmonary function assessment in patients with lung cancer who will undergo surgery (The Japanese Association for Chest Surgery). Gen Thorac Cardiovasc Surg. (2025) 73:385–404. doi: 10.1007/s11748-025-02120-7 39969667

[21] Li X Chen D Yan S Wang Y Wang Y Tao Y . Development and validation of a multi-variable prediction model for major postoperative complications after lung resection in patients aged >/=70 years with non-small-cell lung cancer. J Thorac Dis. (2025) 17:11212–26. doi: 10.21037/jtd-2025-1636 41522131 PMC12780426

[22] Li J Liao C Hu X Luo M Chen C Sun Y . Association between early postoperative hypoxia and postoperative pulmonary complications in lung resection surgery: a retrospective cohort study. J Surg Res. (2025) 315:194–201. doi: 10.1016/j.jss.2025.09.031 41046764

[23] Nagrebetsky A Zhu M Deng H Gaissert HA Gama de. Abreu M, Frendl G, et al. Impaired oxygenation after lung resection: Incidence and perioperative risk factors. J Clin Anesth. (2024) 96:111485. doi: 10.1016/j.jclinane.2024.111485 38718685 PMC11469209

[24] Zhang P Chen Y Xu Z Qin T Yang Y Liu C . Prognostic value of the ARISCAT score for postoperative pneumonia in patients with esophageal squamous cell carcinoma: a retrospective cohort study. Eur J Med Res. (2025) 30:899. doi: 10.1186/s40001-025-03189-9 41024281 PMC12482326

[25] Kotta PA Ali JM. Incentive spirometry for prevention of postoperative pulmonary complications after thoracic surgery. Respir Care. (2021) 66:327–33. doi: 10.4187/respcare.07972 32843511

[26] Franklin E Anjum F. Incentive Spirometer and Inspiratory Muscle Training. Treasure Island, FL: StatPearls Publishing (2025). 34283480

[27] Sweity EM Alkaissi AA Othman W Salahat A. Preoperative incentive spirometry for preventing postoperative pulmonary complications in patients undergoing coronary artery bypass graft surgery: a prospective, randomized controlled trial. J Cardiothorac Surg. (2021) 16:241. doi: 10.1186/s13019-021-01628-2 34429138 PMC8383237

[28] Katsura M Kuriyama A Takeshima T Fukuhara S Furukawa TA. Preoperative inspiratory muscle training for postoperative pulmonary complications in adults undergoing cardiac and major abdominal surgery. Cochrane Database Syst Rev. (2015) 2015:CD010356. doi: 10.1002/14651858.CD010356.pub2 26436600 PMC9251477

[29] Ren J Li Z He Y Gao H Li J Tao J. Systematic review and meta-analysis of breathing exercises effects on lung function and quality of life in postoperative lung cancer patients. J Thorac Dis. (2024) 16:4295–309. doi: 10.21037/jtd-23-1733 39144355 PMC11320241

[30] Wenjie W Yifan J Li W Xiaomin G Linlin Z Yuhan C . Virtual reality-assisted pulmonary rehabilitation enhances early lung function recovery after thoracoscopic surgery in lung cancer patients: a non-concurrent controlled study. Front Med. (2025) 12:1643688. doi: 10.3389/fmed.2025.1643688 PMC12444629 40978740

