# Supplementary material for: A prehabilitation-enhanced nomogram for predicting early pulmonary recovery failure after lung tumor surgery: development and multicenter validation
Source: Front Med (Lausanne). 2026 Jul 13;13:1842606. doi: 10.3389/fmed.2026.1842606 (PMC13402461; doi:10.3389/fmed.2026.1842606)
Supplement: Supplementary file 1 [file Data_Sheet_1.zip › suppplementary material/1Supplementary SOP Incentive Spirometry Target Achieved.docx]

**Standard Operating Procedure (SOP) for Incentive Spirometry Training**

**1. Purpose**

To standardize the delivery, documentation, and quality control of preoperative incentive spirometry (IS) training and to provide an operational definition for the study variable “IS target-achieved days (0–14)”, which quantifies daily adherence during the 14-day prehabilitation window prior to lung tumor surgery.

**2. Scope**

This SOP applies to all eligible patients enrolled in the prehabilitation program during the 14 days before surgery at participating centers. It covers patient education, training schedule, target definition, daily documentation, and quality assurance procedures.

**3. Personnel and Responsibilities**

1. Thoracic surgery nurse / prehabilitation nurse: provides initial education, sets individualized IS targets, reviews daily logs, verifies completion, and records the target-achieved status.
2. Patient (and caregiver, if available): performs IS training as prescribed and completes the daily log (paper or electronic).
3. Site coordinator / research assistant: audits documentation completeness and resolves discrepancies according to predefined rules.

**4. Equipment**

1. Commercial incentive spirometer with volume (mL) markings (volumetric IS device).
2. Training log (paper or electronic form).
3. Optional: pulse oximeter for safety monitoring in high-risk patients.

**5. Safety Screening and Contraindications**

Before initiating IS training, the nurse assesses for severe dyspnea at rest, unstable hemodynamics, acute respiratory distress, uncontrolled chest pain, syncope history during deep breathing, or inability to cooperate. If any safety concern exists, IS training is deferred and the supervising clinician is notified. Training is resumed only after clearance.

**6. Patient Education (Standardized Elements)**

Patients are instructed that IS aims to increase inspiratory capacity, improve alveolar recruitment, and promote effective cough and secretion clearance. Key technique points include slow deep inhalation, brief inspiratory hold, normal exhalation, resting between attempts, and stopping if dizziness or chest discomfort occurs.

**7. Training Procedure (Per Session)**

Position: seated upright (preferred) or semi-recumbent (≥45°).

1. Exhale normally.
2. Seal lips around the mouthpiece.
3. Inhale slowly and deeply to raise the indicator toward the target volume.
4. Hold inspiration for 3–5 seconds (if tolerated).
5. Remove the mouthpiece and exhale normally.
6. Rest 10–15 seconds between breaths to avoid hyperventilation.
7. Repeat for the prescribed number of breaths per session.

**8. Standard Daily Prescription**

Unless otherwise specified by the clinician/nurse due to patient tolerance:

1. Frequency: 3 sessions/day (morning, afternoon, evening).
2. Dose per session: 10 slow maximal inspirations (total 30 inspirations/day).
3. Patients may split sessions (e.g., 5 + 5 inspirations) if fatigue occurs, provided the total daily dose is met.

**9. Target Definition and Individualization**

A “daily target” consists of both dose and quality components.

1. Dose target (mandatory): completion of ≥3 sessions/day and ≥30 inspirations/day in total.
2. Quality target (mandatory): at least 80% of inspirations in a day must meet all of the following criteria: (i) slow and continuous inspiration (no abrupt jerking), (ii) inspiratory hold ≥3 seconds (if tolerated), and (iii) achieved inspiratory volume ≥80% of the individualized target volume.
3. Individualized target volume setting: on Day 1, the nurse measures the patient’s comfortable maximum inspiratory volume (best of 3 attempts). The individualized target volume is set at 80% of that maximum for the first 2 days, then progressively increased as tolerated (e.g., by 100–200 mL every 2–3 days), aiming toward the patient’s achievable maximum without adverse symptoms. Target volume adjustments must be documented.

Note: If a center uses a fixed target (e.g., ≥1500 mL), the individualized target rule should be replaced by the fixed threshold, and the threshold must be reported in the local SOP.

**10. Definition of “Target-Achieved Day (0–14)”**

A day within the 14-day prehabilitation window is counted as a “target-achieved day” if both conditions are met:

1. Dose completed: ≥3 sessions/day and ≥30 total inspirations/day; and
2. Quality met: ≥80% of inspirations reach ≥80% of the individualized target volume with an inspiratory hold ≥3 seconds (unless medically contraindicated).

If either condition is not met, the day is recorded as not achieved (0). If achieved, record as achieved (1). The variable “IS target-achieved days (0–14)” equals the sum of achieved days over the 14-day window (range 0–14).

**11. Documentation Requirements**

Daily documentation must include: date, number of sessions completed, inspirations per session, best inspiratory volume reached each session (mL) or whether target volume was met, symptoms/adverse events (dizziness, chest pain, severe dyspnea), and nurse verification (signature or electronic confirmation). Documentation sources may include patient self-logs with nurse checks or supervised-session nursing records.

**12. Handling of Missing or Partial Records**

1. If a day lacks any documentation and cannot be verified from nursing records, it is coded as not achieved to avoid inflation of adherence.
2. If dose is documented but quality is missing, it is coded as not achieved unless quality can be reasonably verified (e.g., supervised-session record).
3. If a patient is medically advised to pause IS training on a day (documented by clinician/nurse), the day is coded as not achieved, and the reason is recorded for sensitivity analyses.

**13. Quality Assurance and Training**

1. All participating nurses receive standardized training on IS instruction and documentation before study initiation.
2. Site coordinators perform periodic audits (e.g., 10% random sample) to check completeness and consistency of logs.
3. Discrepancies are reconciled by reviewing original nursing notes and patient logs; final coding decisions are documented.

**14. Adverse Event Management**

If dizziness, syncope, chest pain, or severe dyspnea occurs, stop training immediately, allow the patient to rest, reassess SpO₂ if available, and report to the clinician. Resume only after symptoms resolve and clinician approval; adjust dose/target as necessary.
