# Supplementary material for: A prehabilitation-enhanced nomogram for predicting early pulmonary recovery failure after lung tumor surgery: development and multicenter validation
Source: Front Med (Lausanne). 2026 Jul 13;13:1842606. doi: 10.3389/fmed.2026.1842606 (PMC13402461; doi:10.3389/fmed.2026.1842606)
Supplement: Supplementary file 1 [file Data_Sheet_1.zip › suppplementary material/2Supplementary SOP Breathing Training Target Achieved.docx]

**Standard Operating Procedure (SOP) for Breathing Training**

**1. Purpose**

To standardize the delivery, documentation, and quality control of preoperative breathing training and to provide an operational definition for the study variable “Breathing training target-achieved days (0–14)”, which quantifies daily adherence during the 14-day prehabilitation window prior to lung tumor surgery.

**2. Scope**

This SOP applies to all eligible patients enrolled in the prehabilitation program during the 14 days before surgery at participating centers. It covers patient education, training schedule, target definition, daily documentation, and quality assurance procedures.

**3. Personnel and Responsibilities**

1. Thoracic surgery nurse / prehabilitation nurse: provides initial education, demonstrates technique, tailors training targets, reviews daily logs, verifies completion, and records the target-achieved status.
2. Patient (and caregiver, if available): performs breathing training as prescribed and completes the daily log (paper or electronic).
3. Site coordinator / research assistant: audits documentation completeness and resolves discrepancies according to predefined rules.

**4. Equipment**

1. Timer/clock or mobile phone timer for pacing breathing cycles.
2. Training log (paper or electronic form).
3. Optional: pulse oximeter for safety monitoring in high-risk patients.

**5. Safety Screening and Contraindications**

Before initiating breathing training, the nurse assesses for severe dyspnea at rest, unstable hemodynamics, acute respiratory distress, uncontrolled chest pain, syncope history during deep breathing, severe dizziness, or inability to cooperate. If any safety concern exists, breathing training is deferred and the supervising clinician is notified. Training is resumed only after clearance.

**6. Patient Education (Standardized Elements)**

Patients are instructed that breathing training aims to improve ventilation distribution, reduce dyspnea, promote effective cough and secretion clearance, and optimize breathing pattern before surgery. Key technique points include slow nasal inspiration, diaphragmatic (abdominal) expansion, prolonged expiration (especially with pursed lips), brief inspiratory hold when tolerated, resting between cycles, and stopping if dizziness or chest discomfort occurs.

**7. Training Procedure (Per Session)**

Position: seated upright (preferred) or semi-recumbent (≥45°).

1. Warm-up: 3–5 cycles of relaxed tidal breathing.
2. Diaphragmatic breathing: inhale through the nose (about 2–3 seconds) with visible abdominal rise and minimal shoulder elevation; hold 1–2 seconds if tolerated; exhale slowly.
3. Pursed-lip breathing: inhale through the nose (about 2 seconds), then exhale through pursed lips for about 4 seconds (approximately a 1:2 inhalation-to-exhalation ratio), without forced blowing.
4. Thoracic expansion/deep breathing: take 5 slow deep breaths to near-comfortable maximum, with an inspiratory hold of 2–3 seconds if tolerated, then exhale normally.
5. Airway clearance practice (as needed): perform 2–3 gentle huffs (open-glottis forced exhalation) or effective cough techniques, particularly if sputum is present.
6. Rest 30–60 seconds between components to avoid hyperventilation; pause the session if dizziness occurs.

**8. Standard Daily Prescription**

Unless otherwise specified by the clinician/nurse due to patient tolerance:

1. Frequency: 3 sessions/day (morning, afternoon, evening).
2. Dose per session (typical): diaphragmatic breathing 10 cycles; pursed-lip breathing 10 cycles; thoracic expansion 5 cycles; huff/effective cough practice 2–3 times (if indicated).
3. Patients may split a session into shorter blocks (e.g., 5 + 5 cycles) if fatigue occurs, provided the total daily dose is met.

**9. Target Definition and Individualization**

A “daily target” consists of both dose and quality components.

1. Dose target (mandatory): completion of ≥3 sessions/day, and completion of all prescribed components per session (or an approved modified regimen documented by the nurse/clinician).
2. Quality target (mandatory): at least 80% of breathing cycles in a day must meet all of the following criteria: (i) slow, controlled inspiration with diaphragmatic (abdominal) expansion and minimal accessory muscle use; (ii) pursed-lip expiration with an exhalation time approximately ≥2× the inhalation time; (iii) thoracic expansion breaths performed to a comfortable deep-inspiration level with a brief inspiratory hold (≥2 seconds) when tolerated; (iv) no significant adverse symptoms (e.g., severe dizziness, chest pain, marked breathlessness) requiring termination.
3. Individualization: the nurse may adjust cycle counts, inspiratory-hold duration, or component emphasis based on patient tolerance (e.g., prolonged expiration for COPD), while maintaining a structured daily regimen. Any modification and its reason must be documented in the training log.

**10. Definition of “Target-Achieved Day (0–14)”**

A day within the 14-day prehabilitation window is counted as a “target-achieved day” if both conditions are met:

1. Dose completed: ≥3 sessions/day with completion of all prescribed components (or an approved documented modification).
2. Quality met: ≥80% of breathing cycles meet the quality criteria described above (unless medically contraindicated).

If either condition is not met, the day is recorded as not achieved (0). If achieved, record as achieved (1). The variable “Breathing training target-achieved days (0–14)” equals the sum of achieved days over the 14-day window (range 0–14).

**11. Documentation Requirements**

Daily documentation must include:

1. Date, number of sessions completed, and cycles completed for each component.
2. Any modifications to the regimen and the reason (e.g., fatigue, dyspnea).
3. Symptoms/adverse events (dizziness, chest pain, severe dyspnea) and actions taken.
4. Nurse verification (signature or electronic confirmation) when reviewed.
5. Documentation sources may include patient self-logs with nurse checks or supervised-session nursing records.

**12. Handling of Missing or Partial Records**

1. If a day lacks any documentation and cannot be verified from nursing records, it is coded as not achieved to avoid inflation of adherence.
2. If the number of sessions is documented but component completion or quality information is missing, it is coded as not achieved unless it can be reasonably verified (e.g., supervised-session record).
3. If a patient is medically advised to pause breathing training on a day (documented by clinician/nurse), the day is coded as not achieved, and the reason is recorded for sensitivity analyses.

**13. Quality Assurance and Training**

1. All participating nurses receive standardized training on breathing training instruction and documentation before study initiation.
2. Site coordinators perform periodic audits (e.g., 10% random sample) to check completeness and consistency of logs.
3. Discrepancies are reconciled by reviewing original nursing notes and patient logs; final coding decisions are documented.

**14. Adverse Event Management**

If dizziness, syncope, chest pain, or severe dyspnea occurs, stop training immediately, allow the patient to rest, reassess SpO₂ if available, and report to the clinician. Resume only after symptoms resolve and clinician approval; adjust dose/targets as necessary.
