# Supplementary material for: A prehabilitation-enhanced nomogram for predicting early pulmonary recovery failure after lung tumor surgery: development and multicenter validation
Source: Front Med (Lausanne). 2026 Jul 13;13:1842606. doi: 10.3389/fmed.2026.1842606 (PMC13402461; doi:10.3389/fmed.2026.1842606)
Supplement: Supplementary file 1 [file Data_Sheet_1.zip › suppplementary material/3Supplementary SOP Relaxation Training Completed Days.docx]

**Standard Operating Procedure (SOP) for Relaxation Training**

**1. Purpose**

To standardize the delivery, documentation, and quality control of preoperative relaxation training and to provide an operational definition for the study variable “Relaxation training completed days (0–14)”, which quantifies daily completion during the 14-day prehabilitation window prior to lung tumor surgery.

**2. Scope**

This SOP applies to all eligible patients enrolled in the prehabilitation program during the 14 days before surgery at participating centers. It covers patient education, training schedule, completion criteria, daily documentation, and quality assurance procedures.

**3. Personnel and Responsibilities**

1. Thoracic surgery nurse / prehabilitation nurse: provides initial education, recommends an appropriate relaxation modality, ensures patient understanding, reviews daily logs, verifies completion, and records the completion status.
2. Patient (and caregiver, if available): performs relaxation training as prescribed and completes the daily log (paper or electronic).
3. Site coordinator / research assistant: audits documentation completeness and resolves discrepancies according to predefined rules.

**4. Materials**

1. Standardized audio-guided relaxation scripts (hospital-approved) or printed instruction sheets.
2. Timer/clock or mobile phone timer.
3. Training log (paper or electronic form).
4. Optional: quiet room/earphones/eye mask to reduce distractions.

**5. Safety Screening and Contraindications**

Before initiating relaxation training, the nurse screens for acute psychiatric instability (e.g., acute psychosis, severe agitation), active suicidal ideation requiring urgent intervention, or any condition where relaxation training may be inappropriate without clinician input. Patients with severe dizziness, syncope, or uncontrolled chest pain at rest should defer training until medically stabilized. If any safety concern exists, relaxation training is deferred and the supervising clinician is notified.

**6. Patient Education (Standardized Elements)**

Patients are informed that relaxation training aims to reduce preoperative anxiety and stress responses, improve sleep quality, facilitate adherence to prehabilitation, and enhance perioperative coping. Key instructions include selecting a quiet setting, maintaining a comfortable posture, focusing on breathing/body sensations, and stopping the session if distressing symptoms occur.

**7. Training Procedure (Per Session)**

Recommended environment: quiet, comfortable, and safe; avoid performing sessions while driving or operating machinery.

1. Position: seated upright with back support or lying supine; loosen tight clothing.
2. Warm-up: 1–2 minutes of relaxed nasal breathing.
3. Choose one standardized modality per session (preferred): (i) guided mindfulness breathing, (ii) progressive muscle relaxation (PMR), or (iii) guided imagery.
4. Mindfulness breathing (example): focus attention on slow nasal inhalation and prolonged exhalation; acknowledge distractions and gently return attention to breathing.
5. Progressive muscle relaxation (example): systematically tense (5 seconds) and relax (10–15 seconds) major muscle groups (hands/arms/shoulders/face/chest/abdomen/legs), avoiding painful contractions.
6. Guided imagery (example): follow a standardized audio script to visualize a calming scene while maintaining slow breathing.
7. Cool-down: 30–60 seconds of relaxed breathing; rise slowly to avoid dizziness.

**8. Standard Daily Prescription**

Unless otherwise specified by the clinician/nurse due to patient tolerance:

1. Frequency: 1 session/day (recommended in the evening) during the 14-day prehabilitation window.
2. Duration: 10–15 minutes/session (minimum effective duration: 10 minutes).
3. If the patient experiences difficulty sleeping or high anxiety, an additional daytime session may be recommended; completion is still counted if the minimum daily criteria are met.

**9. Completion Criteria and Individualization**

A “completed session” requires both dose and quality components.

Dose component (mandatory): a continuous relaxation training session lasting ≥10 minutes, performed on the given day, using a standardized hospital-approved script or instruction.

Quality component (mandatory): the patient completes the session without premature termination due to distressing symptoms, and reports acceptable tolerance (no severe dizziness, chest pain, panic, or marked breathlessness). Minor interruptions (e.g., brief pause <1 minute) are allowed if the total continuous training time remains ≥10 minutes.

Individualization: the nurse may tailor the modality (mindfulness/PMR/imagery) and posture based on patient preference, COPD-related dyspnea, pain, or mobility limitations. Any changes should be documented.

**10. Definition of “Completed Day (0–14)”**

A day within the 14-day prehabilitation window is counted as a “relaxation training completed day” if the patient completes at least one qualifying session meeting both dose and quality components:

1. Dose completed: ≥10 minutes of standardized relaxation training on that day.
2. Quality met: session completed with acceptable tolerance and no clinically significant adverse symptoms requiring termination.

If the criteria are met, record the day as completed (1). If not met, record as not completed (0). The variable “Relaxation training completed days (0–14)” equals the sum of completed days over the 14-day window (range 0–14).

**11. Documentation Requirements**

Daily documentation must include:

1. Date, modality used (mindfulness/PMR/imagery), and session duration (minutes).
2. Session time (e.g., evening) and setting (home/ward).
3. Tolerance and any adverse symptoms (dizziness, chest pain, panic, severe dyspnea) and actions taken.
4. Nurse verification (signature or electronic confirmation) when reviewed.

Documentation sources may include patient self-logs with nurse checks, or supervised-session nursing records.

**12. Handling of Missing or Partial Records**

1. If a day lacks any documentation and cannot be verified from nursing records, it is coded as not completed to avoid inflation of adherence.
2. If duration is missing or <10 minutes, it is coded as not completed unless verifiable evidence supports meeting the criteria.
3. If a patient is medically advised to pause relaxation training on a day (documented by clinician/nurse), the day is coded as not completed, and the reason is recorded for sensitivity analyses.

**13. Quality Assurance and Training**

1. All participating nurses receive standardized training on relaxation training instruction and documentation before study initiation.
2. Site coordinators perform periodic audits (e.g., 10% random sample) to check completeness and consistency of logs.
3. Discrepancies are reconciled by reviewing original nursing notes and patient logs; final coding decisions are documented.

**14. Adverse Event Management**

If dizziness, chest pain, panic, or severe dyspnea occurs, stop the session immediately, allow the patient to rest, reassess SpO₂ if available, and report to the clinician. Resume only after symptoms resolve and clinician approval; consider switching to a shorter session or a different modality.
