# Supplementary figures and images for: A prehabilitation-enhanced nomogram for predicting early pulmonary recovery failure after lung tumor surgery: development and multicenter validation
Source: Front Med (Lausanne). 2026 Jul 13;13:1842606. doi: 10.3389/fmed.2026.1842606 (PMC13402461; doi:10.3389/fmed.2026.1842606)

**
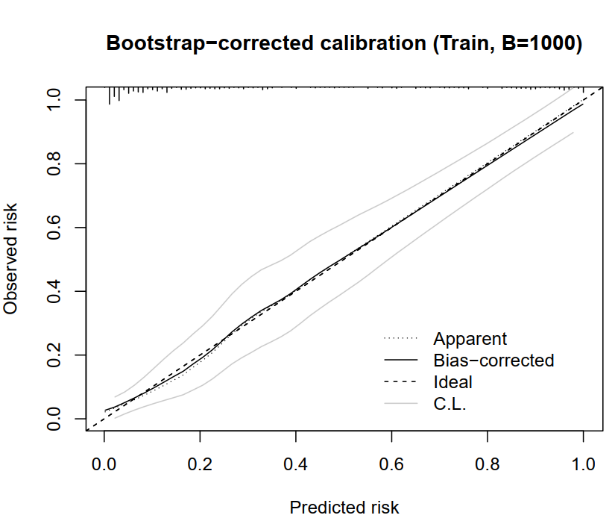
**

**Figure S1 Bootstrap-corrected calibration plot of the nomogram model in the training cohort (B = 1000).**

Supplement: Supplementary file 1 [file Data_Sheet_1.zip › suppplementary material/Supplementary Figures and tables.docx]
